# Immune Privilege as an Intrinsic CNS Property: Astrocytes Protect the CNS against T-Cell-Mediated Neuroinflammation

**DOI:** 10.1155/2013/320519

**Published:** 2013-08-20

**Authors:** Ulrike Gimsa, N. Avrion Mitchison, Monika C. Brunner-Weinzierl

**Affiliations:** ^1^Institute of Behavioural Physiology, Leibniz Institute for Farm Animal Biology, Wilhelm-Stahl-Allee 2, 18196 Dummerstorf, Germany; ^2^Division of Infection and Immunity, University College London, Cruciform Building, Gower Street, London WC1 6BT, UK; ^3^Experimental Pediatrics, University Hospital, Otto-von-Guericke University Magdeburg, Leipziger Straße 44, 39120 Magdeburg, Germany

## Abstract

Astrocytes have many functions in the central nervous system (CNS). They support differentiation and homeostasis of neurons and influence synaptic activity. They are responsible for formation of the blood-brain barrier (BBB) and make up the glia limitans. Here, we review their contribution to neuroimmune interactions and in particular to those induced by the invasion of activated T cells. We discuss the mechanisms by which astrocytes regulate pro- and anti-inflammatory aspects of T-cell responses within the CNS. Depending on the microenvironment, they may become potent antigen-presenting cells for T cells and they may contribute to inflammatory processes. They are also able to abrogate or reprogram T-cell responses by inducing apoptosis or secreting inhibitory mediators. We consider apparently contradictory functions of astrocytes in health and disease, particularly in their interaction with lymphocytes, which may either aggravate or suppress neuroinflammation.

## 1. Introduction

Within the central nervous system (CNS), astrocytes are the most abundant cells. Their main task is to maintain the physiological homeostasis of neurons by providing a stable microenvironment and growth factors. Astrocytes form multicellular syncytia *in vivo* that ensure neuronal homeostasis by taking up excess neurotransmitters and buffering the ionic content of the extracellular medium in the brain. Astrocyte membranes contain numerous neurotransmitter receptors and transporters and can therefore sense and regulate formation, stability, and efficacy of synapses [1]. Recently, they have been shown to play a role in synaptic activity and regulating neuronal circuitry [2–4]. 

**Figure 1:**
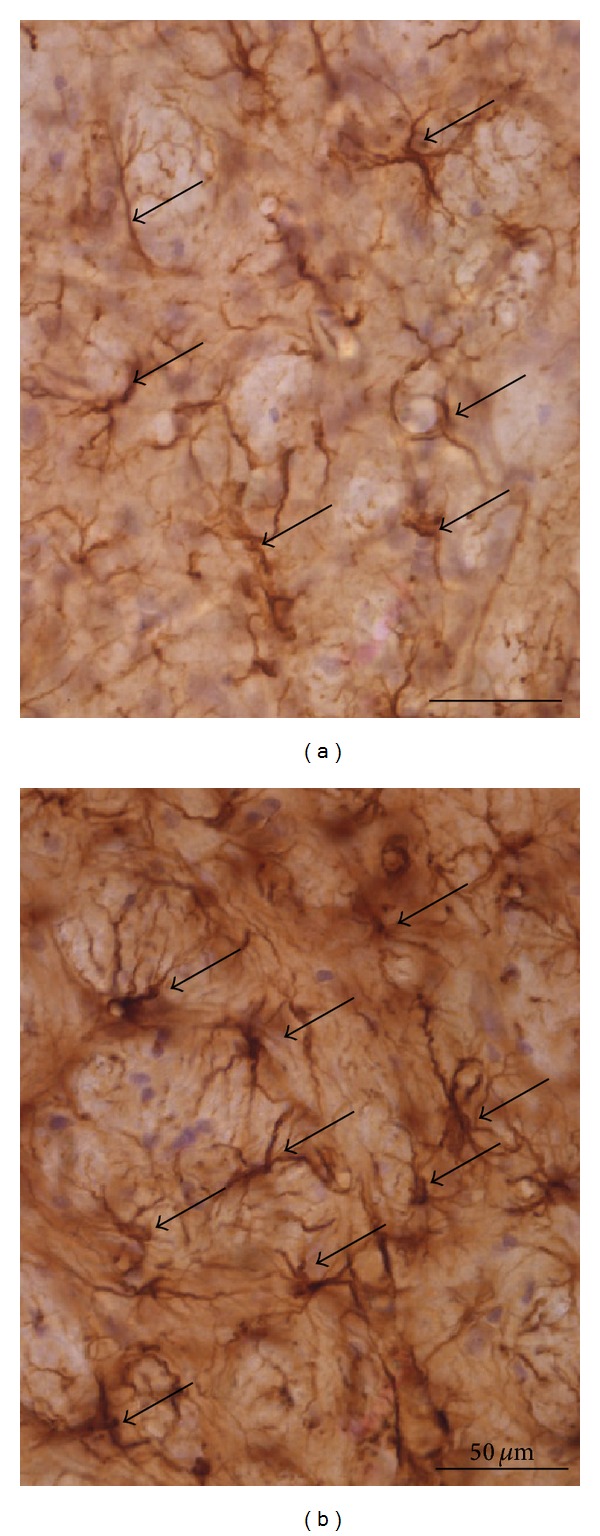
Astrocytes activated in a rat model of Parkinson's disease. Astrocytes (arrows) in the globus pallidus of rats after unilateral striatallesion of dopaminergic neurons by injection of 6-hydroxydopamine (6-OHDA). (a) Contralateral hemisphere; astrocytes have short cellular processes. (b) Ipsilateral hemisphere; astrocytes are in an activated state characterised by long cellular processes and enlarged cell bodies with an intense staining. Staining of glial fibrillary acidic protein (GFAP). For detailed information, see [10].

Astrocytes are dysfunctional in various neurological disorders such as epilepsy, amyotrophic lateral sclerosis, hepatic encephalopathy, stroke, and focal cerebral ischaemia (reviewed in [5]). Dysfunction is often accompanied by astrocytic hypertrophy and an increased number of astrocytic processes, termed astrogliosis [6]. Astrocytes also show these signs of activation in Alzheimer's disease [7, 8] and in Parkinson's disease [9] as well as in its rat model (Figure 1) [10]. Massive astrogliosis has been observed in postmortem tissue of Parkinsonian patients [9, 11–13]. These tissues demonstrated a lack of astrocyte-derived neurotrophins compared to control brains [14, 15]. Because astrocytes support and protect dopaminergic neurons *in vitro* [16], a functional failure of astrocytes may contribute to CNS pathology.

The potential for antigen presentation and production of proinflammatory cytokines by astrocytes has been studied in the neuroinflammatory disease multiple sclerosis (MS) and its animal model experimental autoimmune encephalomyelitis (EAE). They can protect against neuroinflammation by T cells invading the CNS. Thus, they contribute to the immune privilege of the CNS. The privilege is not simply the absence of immune reactions but rather a complicated network of passive and active barriers and of brain tissue. It can modify immune reactions in the CNS so as to minimize the danger of destructive side effects in a tissue with limited ability to regenerate [17]. In this review, we focus on astrocyte functions in health and disease, particularly on their interaction with lymphocytes. 

## 2. Functions of Astrocytes at the Blood-Brain Barrier (BBB)

The BBB limits exchange of solutes between capillaries and the brain parenchyma. Brain capillaries are about 50 to 100 times tighter than peripheral capillaries. This is achieved by complex tight junctions. Astrocytes influence tightness of the BBB by soluble factors that affect endothelial cells [18]. The perivascular space is separated from the brain parenchyma by the basement membrane and the glia limitans, made up of astrocytic end-feet, reviewed in [19]. Notably, it is not the direct contact of astrocytic end-feet with endothelial cells that induces the tightness but soluble factors secreted by them. The presence of numerous astrocytic end-feet close to the BBB allows for a rapid regulation of BBB permeability. Humoural agents that are able to increase BBB permeability and may be secreted by astrocytes include endothelin-1, glutamate, interleukin- (IL-) 1*β*, IL-6, tumour necrosis factor (TNF), macrophage inflammatory protein- (MIP-) 2, and nitric oxide [20]. Soluble astrocytic factors that induce tight junction formation at the BBB are less well characterized. A recent study has shown that sonic hedgehog, a member of the hedgehog signalling pathway family, is produced by astrocytes. Sonic hedgehog promotes BBB formation and integrity, and hedgehog-mediated signals induce immune quiescence in the CNS [21]. Thus, inhibition of hedgehog signalling exacerbates EAE by increasing demyelination, accumulation of leukocytes in the CNS, and production of interferon- (IFN-) *γ* and IL-17 by infiltrating T cells [21].

## 3. Pro- and Anti-Inflammatory Mediators Produced by Astrocytes

Astrocytes are capable of producing a range of proinflammatory cytokines that have been found in the brain of Alzheimer's disease patients such as IL-1*α*, IL-1*β*, IL-6, and TNF [22]. It has been shown that amyloid-*β*_25–35_ in combination with bacterial cell wall lipopolysaccharide (LPS) induced a strong astrocytic production of IL-6 and TNF while neither of the substances alone did [23]. Others found that LPS induced the production of TNF, IL-6, and IL-1 in microglia but not in astrocytes while astrocytes responded neither to LPS nor TNF but to IL-1*β* by producing TNF and IL-6 [24]. This indicates that astrocytes may be regulated by microglial IL-1*β*. Microglial cells produce free radicals and proinflammatory cytokines such as TNF-*α* when exposed to amyloid-*β*_1–42_ [25, 26]. TNF and superoxide anion production by macrophages cocultured with amyloid-*β*_1–42_ was strongly reduced in the presence of primary human astrocytes or astrocytoma cells. Interestingly, astrocytes bound amyloid-*β*_1–42_ and showed activation of the transcription factor NF*κ*B in that study, but unlike in macrophages this activation did not result in TNF production. This indicates that distinct signal transduction pathways are activated in macrophages and astrocytes by inflammation [27]. Indeed, astrocytes can also downregulate microglial activation by the secretion of anti-inflammatory substances such as transforming growth factor- (TGF-) *β* and prostaglandin E_2_ (PGE_2_) [28, 29] and may thereby limit inflammation-induced neurodegeneration. However, activated microglia can also reduce amyloid-*β* accumulation by phagocytosing and degrading it [30]. Thus, the clinical relevance of both astrocytic and microglial activation has not yet been fully elucidated. 

Glia maturation factor (GMF) is produced by astrocytes. It is not only necessary for the growth and maturation of neurons and glia cells, but can also induce the production of proinflammatory cytokines. Overexpression of GMF in astrocytes induces the production and secretion of granulocyte-macrophage-colony stimulating factor (GM-CSF), an activation of microglia and the expression of proinflammatory genes including major histocompatibility complex- (MHC-) II, IL-1*β*, and MIP-1*β* [31]. Knockdown of GMF reduces the production of the proinflammatory cytokines and chemokines responsible for EAE [32, 33]. Interestingly, it also inhibits growth of glioblastoma cells by inducing G0/G1 cell cycle arrest *in vitro* [34, 35]. In the brain of Alzheimer's disease patients, GMF is upregulated [36, 37]. However, what drives astrocytes to upregulate GMF to a level where it contributes to tissue damage is unknown.

Astrocytes produce or take up, store, and reexocytose a range of neurotrophins neuroprotective in EAE [38–41], dementia of the Alzheimer type [42], and Parkinson's disease [43, 44]. Astrocytes are the major source of nerve growth factor (NGF) and glial cell line-derived neurotrophic factor (GDNF) in the CNS [45–47]. In brain tissue of Parkinson's disease patients, GDNF, NGF, and brain-derived neurotrophic factor (BDNF) are deficient [14, 15], hence the clinical trials of therapeutic GDNF injection into the brain of Parkinson's patients. While intraputaminal infusion of GDNF was safe and improved motor functions in a small group of patients over one [48] and two years [49], a randomized placebo-controlled trial found that motor function has not improved [50]. Notably, from all the 32 genes associated with astrocyte function described in this review, only GDNF was found to be associated with a disease: “major depressive disorder.” For this, see the NCBI catalog of genomewide association studies (GWAS) (http://www.genome.gov/gwastudies/).

On the other hand, as mentioned above, astrocytes are a major source of the proinflammatory cytokines IL-1*β* and IL-6 in the brain [51, 52]. Transgenic mice that lack IL-6 production are resistant to EAE induction [53, 54]. This is due to a blockade of activation and differentiation of autoreactive T cells in the periphery with both T helper (Th) 1 and Th2 cells differentiation being affected [53]. Very recently, dendritic cells have been identified as a sufficient and probably the main source for EAE induction [55]. Whether astrocytic IL-6 plays a decisive role in the etiogenesis of EAE has been ruled out in animal models. Transgenic mice that overexpress IL-6 in astrocytes but are otherwise deficient in IL-6 develop a mild form of ataxia, but no symptoms of lymphocyte-driven EAE. These mice had indeed cellular infiltrates in the cerebellum independent of MOG immunisation [56]. Thus, the observed ataxia may be a result of a general inflammatory process in the brain.

It is known that IL-1*β* plays an important role in MS and EAE. Families with a high IL-1*β* over IL-1 receptor antagonist (IL-1Ra) production ratio have a higher risk to have a patient relative with MS than families with a low ratio [57]. Mice deficient in IL-1 receptor type I (IL-1RI−/−) are resistant to EAE induction [58, 59]. Apparently, IL-1*β* is necessary for the induction of IL-17-producing T cells (Th17) [59]. IL-17 has been shown to be crucial for the development of EAE [60, 61]. However, both IL-6 and IL-1*β* do not necessarily have only detrimental effects. Recently, IL-6 has been demonstrated to induce IL-10 in T cells and thus inhibit proinflammatory responses of Th1 cells [62]. The production of IL-1*β* and IL-6 does not necessarily lead to neuronal damage because these cytokines also induce upregulation of Fas ligand (FasL) in astrocytes, which may induce T-cell apoptosis [63] (see below). In addition, IL-1*β* and IL-6 are messengers between the brain, particularly the hypothalamic-pituitary-adrenal axis, and the immune system. Thus, IL-1*β* produced during EAE upregulates glucocorticoid production which has a downregulatory effect on inflammation [64]. 

## 4. Interactions of Astrocytes and T Lymphocytes

### 4.1. Induction of Apoptosis in Activated T Cells

Activated T cells can cross the BBB not only in neuroinflammatory diseases but also in the healthy brain [65, 66]. Later, it has been shown that in macrophage-depleted mice, activated T cells which extravasate are not able to enter the brain parenchyma via the basement membrane but accumulate in the perivascular spaces [67]. Matrix metalloproteinases (MMP-) 2 and -9 are necessary to cross the basement membrane after local digestion [68]. These enzymes could be produced by perivascular macrophages.

 These infiltrating T cells may combat infection, but damage to tissue needs to be avoided, and in particular that mediated by Th1 and cytotoxic T cells and accompanied by inflammation. Inflammatory cytokines such as TNF-*α* are neurotoxic. Given that neurons have a very limited capacity to regenerate in the mature brain, side effects could be detrimental. One mechanism preventing damage is elimination of T cells: astrocytes induce apoptosis in these cells [69–71]. This effect is mediated by the expression of FasL (CD95L) by astrocytes [63, 72, 73]. In EAE, FasL expressing astrocytes exist in close vicinity to apoptotic T cells [74, 75]. The same mechanism of enforcing immune-privilege has been observed in placenta [76–79], testes [80], and anterior chamber of the eye [81]. A downside of this mechanism is that astrocytoma express FasL and thus escape immune attack [82, 83].

### 4.2. Astrocytes as Antigen-Presenting Cells in Neuroinflammation

In neuroinflammation, astrocytes can act as antigen-presenting cells (APCs) [84, 85]. While microglia express MHC-II readily upon activation *in vivo* and *in vitro*, astrocyte MHC-II expression occurs only during prolonged inflammation *in vivo* [86] or *in vitro* under stimulation by interferon- (IFN-) *γ* [87]. This MHC-II induction may be suppressed by neurons via a mechanism that has not fully been elucidated. One study claims that cell-cell contact is required [88] while another one found that secreted glutamate and norepinephrine could inhibit IFN-*γ* induced MHC-II expression in astrocytes [89]. In keeping with this, neuronal loss induces MHC-II expression in astrocytes [88, 90], supporting the view that astrocytes can present antigen only during severe neuroinflammation. The expression of costimulatory B7 molecules by astrocytes both *in vivo* and *in vitro* has been controversially discussed. While some authors found B7 expression on astrocytes [91–94], others did not [95, 96]. Functioning as APCs *in vitro*, astrocytes have been found to stimulate differentiated T cells; and interestingly, they stimulate Th2 cells more efficiently than Th1 cells [87, 97]. Th2 cells may be less damaging than the cellular immune responses, and hence the preferred agents of protection against infection in the CNS. Thus, astrocytes from transgenic mice expressing MS-associated MHC-II human haplotypes HLA-DR2 and HLA-DR4 induced a mixed Th1/Th2 cytokine response in MOG-specific T cells, whereas dendritic cells induced a Th1 response [98]. One can only speculate about the biological relevance of an astrocyte-mediated Th2 bias. In EAE, T cells typically enter the CNS as activated, differentiated Th1 cells. However, the T-cell population may not consist exclusively of Th1 cells. If astrocytes preferentially restimulate Th2 cells [87, 97], the proportion of these cells could increase, thus favouring an anti-inflammatory microenvironment. Also, memory T cells are recruited to the CNS during EAE [99]. Memory cells are heterogeneous and part of the population is not biased for a certain Th subpopulation, yet. Thus, it is tempting to speculate that astrocytes may prevent induction of a Th1 cytokine profile of memory cells in the CNS [100]. The astrocyte-mediated bias towards Th2 responses cannot be explained by their cytokine secretion as astrocytes do not produce IL-4, which is the main inductor of Th2 responses, but might rather reflect the signal strength of the MHC-II-T-cell receptor (TCR) interaction. Lowering the signal strength has been found to favour Th2 differentiation [101]. For instance, the surface density of MHC-II expression determines the cytokine profile of T cells with low MHC-II expression levels favouring Th2 responses [102]. Astrocytes do not readily express MHC-II molecules and are thus likely to deliver a weaker TCR signal than “professional” APCs with higher density of MHC-II molecules on their surface. 

### 4.3. Suppression of T-Cell Functions

In EAE, infiltrating T cells do not proliferate in the target organ [103]; this has been ascribed to the influence of astrocytes [104]. *In vitro*, astrocytes can either suppress [105–107] or stimulate [87, 97, 108] T-cell functions. In coculture studies, astrocytes induce hyporesponsiveness in T cells. This was interpreted as a result of downregulation of the TCR [105] and insufficient stimulation by low levels of ICAM-1 on astrocytes [106]; this would limit adhesion of T cells to astrocytes, so that the two cells ignore each other. As this would not silence invading T cells in the CNS, other mechanisms may have been involved which are not fully understood, yet.

T-cell activation is tightly regulated by surface molecules, providing scope for immunotherapy [109–111]. While the primary costimulatory molecule CD28 and its homologue CTLA-4 (cytotoxic T-lymphocyte-associated antigen-4, CD152) on T cells engage the same ligands B7-1 (CD80) and B7-2 (CD86) on APCs, CTLA-4 binds with 10–100-fold higher affinity than CD28 [110, 112]. CD28 signaling initiates, sustains, and enhances T-cell activation while CTLA-4 signaling inhibits T-cell activation and attenuates ongoing responses [110, 113, 114]. The relevance of this has been demonstrated by genetic inactivation of CTLA-4 in mice, which leads to lymphoproliferative disease and early death [110, 112]. T cells of this mouse strain proliferate spontaneously *ex vivo* and show an activated phenotype stressing the central role of CTLA-4 in attenuating unwanted T-cell responses. In contrast to CD28, which is constitutively expressed on the surface of T cells, CTLA-4 is not detectable on resting T cells [114]. Expression of CTLA-4 mRNA and CTLA-4 protein on the T-cell surface is induced upon activation. CTLA-4 is stored intracellularly, and its surface expression is strictly controlled with a peak after 48 h–72 h after T-cell stimulation [114, 115]. Blockade of CTLA-4 in mouse models of autoimmune diseases increases the incidence of EAE [111, 116]. Short blockade of CTLA-4 during priming of the immune response has lasting effects, suggesting that failure in the regulation of CTLA-4 would have long-lasting impact on immune responses including autoimmunity [117]. Thus, giving agonistic CTLA-4 signals might be a promising strategy for controlling inflammatory responses in the CNS, particularly as CTLA-4 is highly expressed on the T cells which accumulate there [118]. 

Our own study showed that astrocytes inhibit T-cell proliferation, production of IL-2 and IL-10, and expression of the IL2R *α*-chain (CD25) [107]. Functionally, astrocytes mediated these effects by upregulating CTLA-4 on Th1 and Th2 cells. Although inhibition did not require astrocyte contact with T cells, the mechanism was independent of the major inhibitory cytokine TGF-*β*. The study provided optimal stimulation for T cells by having professional APCs and antigen in the cultures when astrocytes were added. Thus, astrocytic inhibitory or stimulatory effects could be discerned from baseline effects occurring during T cell-APC interaction. In this way, we also avoided differences in the stimulatory capacity of astrocytes towards Th1 versus Th2 cells [87, 97]. The interpretation is supported by a recent study showing that astrocytes inhibited proliferation and IFN-*γ*, interleukin- (IL-) 4, IL-17, and TGF-*β* secretion levels of encephalitic T cells *in vitro* unless they were pretreated with IFN-*γ*. They even promoted T-cell proliferation, presumably by additional antigen presentation [119]. The inhibitory effect of astrocytes could be ameliorated by IL-27 neutralisation [119]. IL-27 has been shown to suppress Th17 cells and thereby EAE [120, 121]. Also, it negatively regulates Th17 cells during chronic inflammation of the CNS resulting from chronic infection with *Toxoplasma gondii* [122]. Coculture of astrocytoma cell lines with CD3/CD28-activated T cells revealed suppression of T-cell proliferation. The effect was more pronounced when direct contact was allowed between astrocytes and T cells but remained strong when astrocytes and T cells were separated by cell culture inserts [123]. The finding that T-cell proliferation was still inhibited by astrocytes when astrocytes and T cells were separated by a cell culture insert or a transwell-membrane showed that a soluble factor produced by astrocytes is responsible for this inhibition [107, 123, 124]. However, astrocytes might conceivably have protruded cellular nanotubes through the cell culture inserts so as to contact the T cells. The separating membranes had pore sizes of 200 nm [123] or 400 nm [107, 124]. An electron-microscopical study of astrocytes growing on engineered surfaces showed that astrocytes extend nanotubes with a diameter below 100 nm to make contact with other cells and may even exchange substances via these nanotubes [125]. This may be a mechanism which allowed astrocytes to contact the T cells physically. Cell-cell contact did not bear sole responsibility for the control of T-cell proliferation, since astrocyte-conditioned supernatant also inhibited T-cell proliferation [124]. Despite being of interest for immunotherapy, the nature of this soluble inhibitory factor remains unclear. Blockade of TGF-*β* had no [124] or only a minor effect [107] on the inhibition of T-cell proliferation. Inhibition of nitric oxide production also did not reverse the inhibitory effect [123, 124]. Furthermore, inhibition of indoleamine-2,3 dioxygenase (IDO) by methyltryptophan did not affect astrocyte-mediated inhibition of T-cell proliferation [123].

IDO is a tryptophan-degrading enzyme and as such inhibits T-cell proliferation. It has been proposed as a major player in the immune privilege of the placenta [126]. Astrocytes and microglia are capable of expressing IDO *in vitro* and *in vivo* upon activation with IFN-*γ* [127]. IDO blockade in EAE mediates disease exacerbation, suggesting that IDO induction by Th1-derived IFN-*γ* may play a role in self-limiting autoimmune inflammation during EAE and MS [128]. IDO can also induce tolerance of tumours in the CNS [129]. PGE_2_ induces IDO in dendritic cells [130, 131]. Systemic administration of cytosine-phosphate-guanine dinucleotide (CpG), a frequent dinucleotide in bacterial DNA and therefore detected by pattern recognition receptor Toll-like receptor-9 (TLR-9), upregulates IDO in plasmacytoid dendritic cells, where it is required for activation of regulatory T cells (Tregs), and blocks their conversion into Th17 cells [132]. Although likely, whether IDO induction in astrocytes by PGE_2_ or CpG plays a role in the CNS and whether astrocytes can induce Treg activation is one of the open questions concerning astrocytes so far. IDO-deficient mice develop exacerbated EAE with enhanced Th1 and Th17 responses [133]. In this model, not only tryptophan depletion was responsible for the effect on T cells but also a downstream tryptophan metabolite from the kynurenine pathway, 3-hydroxyanthranilic acid (3-HAA), was. The kynurenine pathway starts with tryptophan degradation by IDO or tryptophan-2,3 dioxygenase (TDO) leading to 3-HAA. 3-HAA was shown to increase the percentage of Tregs and inhibited Th1 and Th17 cells leading to EAE amelioration [133]. 3-HAA has been shown to be neuroprotective in cytokine-mediated inflammation *in vitro* [134] while other metabolites of the kynurenine pathway such as 3-hydroxykynurenine and quinolinic acid (QUIN) appear to be neurotoxic [135]. Another metabolite of the IDO-kynurenine pathway is kynurenic acid (KYNA) which has been shown to be neuroprotective [136]. Interestingly, activated human astrocytes have been shown to produce large amounts of KYNA but almost no QUIN [137]. Thus, astrocytic IDO activation may lead to various effects which are mostly beneficial. 

Astrocytes in a rat EAE model could induce development of Tregs, as has been shown in a study where T cells that had been cocultured with astrocytes not only lost ability to proliferate and inhibit proliferation of antigen-stimulated T cells but also markedly alleviated the disease [138]. Also in this study a heat-sensitive soluble factor was implicated, other than IL-10 or TGF-*β* [138].

Another surface molecule, B7-H1 (PD-L1), might downregulate T-cell responses in the CNS; it is a member of the B7-family known to downmodulate T-cell activity [139]. In a model of fiber tract injury in the hippocampus of adult mice, it is strongly upregulated on astrocytes while T-cell recruitment to the site of injury was not accompanied by autoimmune demyelination [140].

### 4.4. Astrocyte-Released Signals That May Influence T-Cell Influx

Astrocytes are efficiently activated by the IFN-*γ* produced by Th1 cells (see above). Under the influence of IFN-*γ*, astrocytoma cells upregulate expression of chemokines including CCL3, CCL5, CXCL8, and CXCL10, as well as proinflammatory cytokines such as IL-6 and IL-1*β* (but also an anti-inflammatory IL-1 receptor antagonist) [123]. Most of these chemokines attract Th1 cells more than Th2 cells, thus aggravating neuroinflammation. Thus, astrocytes may inhibit and delay neuroinflammation, but in case of sustained inflammation accompanied by high IFN-*γ* levels, they may switch to become potent APCs and even promotors of inflammation [119].

### 4.5. T-Cell-Mediated Induction of Nerve Growth Factor

Nerve growth factor (NGF) is a member of the neurotrophin family. Growth, differentiation, survival, and maintenance of peripheral and central neurons are facilitated by NGF [143]. NGF administered intracerebroventricularly into marmosets delays the onset of EAE and reduces lesion formation [41]. Subsequent to induction of EAE, mice treated with NGF by intraperitoneal injection exhibited a delayed onset of disease in combination with lower clinical disease scores [144]. Moreover, myelin basic protein- (MBP-) specific T cells retrovirally transduced to secrete high levels of NGF are unable to mediate clinical EAE and suppress induction of EAE by nontransduced MBP-specific T cells in rats [40]. Astrocyte-T-cell interaction results in increased NGF production by astrocytes. This upregulation was found to be dependent on antigen recognition as blockade of MHC-II abrogated the effect, and resting astrocytes which were not able to present antigens did not show an upregulation of NGF production. Neutralisation of the cytokines IFN-*γ*, IL-4, and IL-10 produced in the cocultures did not affect NGF production [142]. This finding suggests a neuroprotective role of astrocytes during T-cell-mediated inflammation in the CNS. Conversely, cells of the immune system carry NGF receptors, and NGF signalling modulates immune function. Perivascular infiltrates of NGF-treated marmosets decrease IFN-*γ* and increase IL-10 expression [145]. NGF inhibits the MHC-II inducibility of microglia, thereby limiting antigen-presentation in the CNS [146].

**Figure 2:**
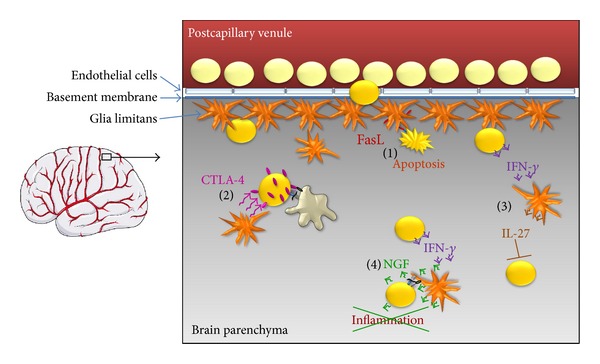
Astrocytes enforce the immune privilege of the CNS (left) at multiple checkpoints employing various mechanisms (right). Astrocytes in the glia limitans are responsible for the exceptional tightness of endothelial tight junctions by producing soluble factors [18]. Despite the BBB, activated T cells (yellow) are able to enter the brain parenchyma (grey) [65]. (1) At the same time, astrocytes in the glia limitans and in the parenchyma may express FasL while activated T cells may express Fas [63, 72, 73]. The ligation of Fas and FasL induces apoptosis of T cells [71]. (2) As this does not fully eradicate infiltrating T cells, the surviving T cells may be restimulated by activated microglia presenting CNS-specific antigens on MHC-II. In the presence of astrocytes, T cells upregulate CTLA-4 [107] which upon ligation of B7 molecules induces a stop of proliferation and anergy of the T cells. (3) IFN-*γ* produced by invading T cells stimulates astrocytic IL-27 production which suppresses Th17 cells [120, 121, 141]. (4) During sustained T-cell-mediated inflammation, IFN-*γ* secreted by T cells activates astrocytes to gain the ability to present antigen on MHC-II and costimulate T cells. While this cognate interaction may exacerbate neuroinflammation, it simultaneously leads to an upregulation of NGF production that counteracts neuroinflammation [142]. Also, astrocytes acting as APCs appear to promote Th2 responses and the formation of regulatory T cells [138]. Astrocytes: orange cells; pink: effects leading to CTLA-4 upregulation; green: effects of NGF; dark red: blood; grey: brain parenchyma.

Mechanisms by which astrocytes maintain immune privilege or limit inflammation-induced damage are summarised in Figure 2.

## 5. Conclusions

For a long time, the CNS has been considered immune-privileged. However, the initial explanation of a strictly sealed BBB weakened when activated T cells were found to cross the BBB in the healthy brain. Clearly, various cells contribute to the phenomenon, including astrocytes, the most abundant cells of the CNS. Astrocytes mediate neuronal differentiation and homeostasis, and evidence is increasing that astrocytes interact with the immune system. The concept of immune privilege of the CNS may be weakening, but it is clear that astrocytes dampen inflammation and have beneficial, neuroprotective effects on the healthy brain. Astrocytes need activation by IFN-*γ* to unfold their anti-inflammatory potential, in forms such as IL-27 production [141]. Even when unable to prevent T-cell responses in the brain after prolonged provocation (e.g., by IFN-*γ*), their function does not become purely detrimental. When activated, astrocytes harbour mechanisms of damage limitation, such as production of neuroprotective NGF and preferential restimulation of Th2 over Th1 cells. When this is not sufficient to prevent autoimmune damage to the CNS, it may still control tissue damage to some extent. The overall picture of astrocytes is as CNS-intrinsic cells that combat local inflammation and maintain immune privilege, thus minimising damage.
